# Ultra-thin broadband solar absorber based on stadium-shaped silicon nanowire arrays

**DOI:** 10.1007/s12200-022-00010-x

**Published:** 2022-04-06

**Authors:** Seyedeh Leila Mortazavifar, Mohammad Reza Salehi, Mojtaba Shahraki, Ebrahim Abiri

**Affiliations:** 1 Department of Electrical and Electronics Engineering, Shiraz University of Technology, Modarres Blvd, 71557-13876 Shiraz, Iran; 2 Faculty of Electrical and Electronics Engineering, University of Sistan and Baluchestan, Daneshgah Blvd, 98613-35856 Zahedan, Iran

**Keywords:** Ultra-thin solar cells (SCs), Light trapping, Stadium silicon nanowire (NW), Optical resonators, Diffraction

## Abstract

This paper investigates how the dimensions and arrangements of stadium silicon nanowires (NWs) affect their absorption properties. Compared to other NWs, the structure proposed here has a simple geometry, while its absorption rate is comparable to that of very complex structures. It is shown that changing the cross-section of NW from circular (or rectangular) to a stadium shape leads to change in the position and the number of absorption modes of the NW. In a special case, these modes result in the maximum absorption inside NWs. Another method used in this paper to attain broadband absorption is utilization of multiple NWs which have different geometries. However, the maximum enhancement is achieved using non-close packed NW. These structures can support more cavity modes, while NW scattering leads to broadening of the absorption spectra. All the structures are optimized using particle swarm optimizations. Using these optimized structures, it is viable to enhance the absorption by solar cells without introducing more absorbent materials.

**Graphical Abstract:**

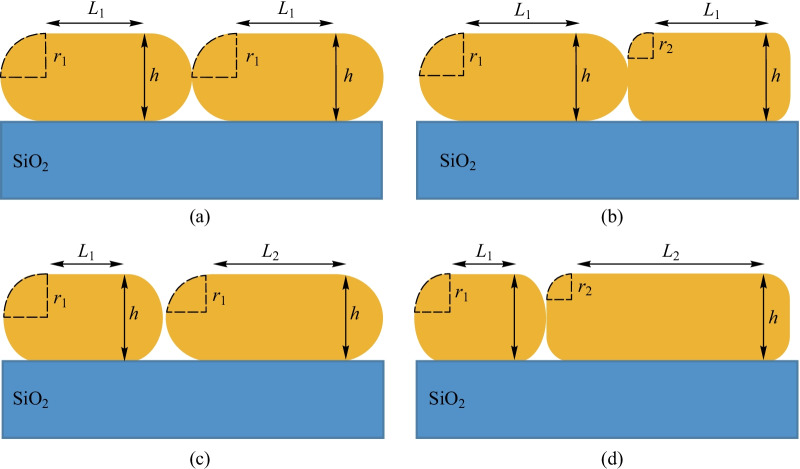

## Introduction

Solar energy can be considered the best source of renewable, abundant, non-concentrated, and non-polluting energy. Solar cells attract the attention of scientific researchers due to advantages including profitability, low weight, pliability, and ease of construction. Among all energy sources, solar cells (SCs) is not the cheapest. However, since the SC is known to be a key source of sustainable electricity generation, there is a need to improve its efficiency and cost effectiveness. Silicon-based SCs have proved effective yet they are expensive to manufacture. Promising approaches to minimization of light reflectivity and improvement of solar absorption include the use of metallic nanoparticles [1]; transparent nanostructured electrodes [2, 3]; and various microstructures and nanostructures as anti-reflective coatings (ARC), such as pyramids [4], rectangular grooves [5], pillars [6], nanowires (NWs) [7, 8] and nano-cones [9].

In a NW, a quantum (wave) mechanical interpretation is more applicable than a classical one. The quasi-three-dimensional NW nanostructure has fantastic optical, electrical and mechanical performance, which makes it very appealing for use as transparent electrodes in modern photovoltaics (PVs) [10], lasers [11], sensors [12], light-emitting diodes [13], and thin films [14]. All of these applications are made possible by highly optimized optical resonators which can efficiently capture electromagnetic energy in narrow frequency bands. In practice, for semiconductor NW-PVs, e.g., in SCs [15], the improvement of light absorption efficiency is crucial in order to maximize the performance. Therefore, great efforts have been made to find an ideal structure for a NW array.

NW-PVs have unconventional light absorption properties due to the fact that in conventional cavities there is a simple compromise between bandwidth and enhancement of the trapped energy: the greater the enhancement is, the narrower will be the bandwidth. This is a unique feature of subwavelength cavity.

In a NW cavity, the optical resonances which depend on morphology also lead to efficient light absorption across a wide range of wavelengths [16]. Such features highlight the need for further NW cavity design and synthesis that could improve absorption efficiency and short-circuit current density (*J*_SC_); and exceed conventional limits. Subsequently, a more innovative approach has come to the fore of attention, where, for example, a dielectric core–shell structure can balance the anti-reflection effect of dielectric materials and the enhancement of absorption induced by leaky mode resonances [17–19]. Moreover, tests in a different direction have been conducted to manipulate wire array configurations such as square, triangular, Penrose, and random structures [20]. Besides, ample studies focus on designing the grating-structured NWs made from various materials with certain optical properties [21–23].

A basic problem associated with silicon NWs is precisely controlling the size, diameter, placement, and orientation. Once the stadium nanowires (SNWs) are grown, issues such as handling, positioning, aligning, and assembling need to be worked out in the post-growth steps. All these tasks involve using leading edge technology due to the micro size of the NW.

The incompatibility of growth methods with the present silicon thin film industry is a major problem [24]. Nano electronic devices with a vertical structure has not yet been implemented in the industry. When the NW’s length overreaches a certain limit, it breaks and falls horizontally on the substrate. The architecture of nano electronic devices is more compatible with horizontal electronic devices. Therefore, for a one-step fabrication of NW integrated device, three different methods are employed including thin-film-fracture-based for the synthesis of horizontal NWs, vapor liquid–solid (VLS), and the direct oxidation of metal powders for the synthesis of vertical NWs. The thin-film-fracture-based technique has been used for the synthesis of a well-aligned horizontal NW [25].

First introduced in 1964, VLS growth method [26] has gained significant attention during the last decade [27], as it is a means of manufacturing stress-free single-crystal semiconductor NWs with unique electronic and optical properties which are suitable for ultraminiaturized electronics [28], optoelectronics [29] and renewable energy [30]. However, assembly of horizontal NWs on a large scale with intended orientation on diverse surfaces is barrier to their integration into practical devices. To do so, some processes of assembly have been reported such as utilization of liquid flows [31], electric fields [32], Langmuir–Blodgett compression [33], and mechanical shear [34]. VLS growth contributes to the manufacture of NWs with intended (non-) polar crystallographic orientations that impact their optical and electronic characteristics. Epitaxial growth at distinct lattice directions, based on maximum reduction of interfacial energy and strain is the challenge before application of guided VLS growth of horizontal NWs. A horizontal VLS growth method that yields high-quality NWs that are parallel to the surface was developed by the Joselevich group [35].

In addition, NWs can have diameters that are smaller than the wavelength of the light, which enables large scattering in the physical cross-section [36] and localized optical resonances [37]. The research on laterally oriented NWs in PV studies has only targeted single NW devices [38, 39], while vertically oriented NW-PVs have been investigated from both aspects [40]. However, since the device structure and fabrication methods mostly require horizontal geometries, vertical NWs are not necessarily very practical for electronics. This is where the guided growth method can determine the orientation of a NW; if the system is precisely engineered, it will grow along with the established geometries. For the fabrication of integrated circuits, it is of paramount importance to position the NWs in a well-adjusted dense array. By growing the NWs directly into place for each device any further arrangement steps can be avoided.

With an equal thickness of nanowire arrays, the horizontal NW array had a highly efficient performance in comparison to the thin film structure, taking benefit from its light concentration and light trapping properties. This insight could lay the foundation for building ultra-thin, highly efficient SCs using very low-cost materials. A number of nano-patterning techniques are used to perform the horizontal NW fabrication on the Si-on-insulator (SOI) substrates generally [41, 42]. The oxide layer buried in the SOI serves as the etch-stop layer. Although a good size-control can be achieved, these lithography processes are limited by their low productivity for wafer-scale NW fabrication [43]. A series of two-dimensional stadium-shaped planar resonators in planar photonic crystals fabricated in SOI are designed and experimentally realized. A certain geometry can be improved in functionality up to six times by re-designing the shape alone, i.e., without incurring any extra cost or needing more materials. Fabrication of a chaotic system is then less challenging when the tolerances are relaxed. A recent study has shown that when the average decay rate of modes comes to a particular value the energy storage capacity is upgraded, which means better light absorption in chaotic resonators with a stadium cross-sectional shape [44].

Tunneling has been studied in the electrical conductivities of NW networks. When a NW is represented as a hard conductive core with a soft shell, tunneling occurs as the shells belonging to different wires overlap. A core and a stadium shaped shell make a wire that produces tunnel conductivity when two shells overlap [45].

Lieber’s group is one of the pioneers who have placed NW on a micro-stadium structure [46]. Since the vertical stadium silicon NWs have been successfully fabricated and used as an efficient absorbent, crystalline silicon is used as the absorbing material. The process of stadium NW arrays fabrication is described in Refs. [44, 47].

Taking NWs to a definite position sideways and connecting them to the metal electrodes is the main hurdle. The prevalent transfer method primarily has two phases [48, 49]. For the first phase, NWs are distanced from growth substrate and then a volatile solvent is used to disperse them. Next, a single drop of the solvent is cast on the target substrate. For the second phase, lithography helps in determining the electrode windows, followed by metal deposition and lift-off techniques. But in this case NWs are arbitrarily arrayed on the substrate. Obtaining a device with NWs that connect to metal contacts at both ends takes a lot of time. In addition, manufacturing a device with few aligned NWs through the above method is quite challenging. A few methods and certain equipment of assembly of NWs have been reported in detail [34, 50–53]. Manufacture of devices with horizontally arrayed silicon NW has been reported such as [54]:

• a ${\mathrm{SiO}}_{2}$ substrate prepared by thermal oxidization of an n-type wafer is utilized and spin-coating of photoresist is done;
• perpendicular pressing of the etched substrate on intended substrate, using metal-assisted chemical etching (MACE) method [55];
• silicon NWs are transported on intended substrate;
• spin-coating of photoresist’s second layer was performed;
• through photolithography and lift-off processes, multi-wire devices are properly manufactured.

A simple technique of manufacture of uniform horizontal silicon NW arrays with intended orientation and density at spatially well-defined spots on substrate is premised on an in-situ hard mask microphase separated approach [56].

The symmetric supplementation of two half-ellipses to the antithetical sides of a rectangle results in a two-parameter planar domain called elliptical stadium. In the relevant elliptical stadium, the point particle travels at a fixed velocity within this boundary, showing mirror reflections on the walls. This is a general depiction of a Bunimovich stadium with ring-shaped arcs, which remains completely ergodic no matter how long the central rectangle is [57]. Some descriptions of the stadium are included in the literature [58–61]. All potential shapes of boundary and specifications of the stadium are addressed [62, 63]. Experiments on the stadium-shaped microwave resonant cavities might be done to stadia with elliptical arcs, possibly with fascinating results [64].

The common value tends to be regular for the lifetime of all modes in chaotic cavities, as a result of which the higher energy storage capacity in a chaotic resonator can be explained perceptively by choosing an RO-based (ray-optics) approach, taking into account that a good shape deformation comes with a break in symmetric structure. As a result, the deformed resonator cannot withstand cyclic motion of light, and the path of the lights will change from regular to random. The cause of the chaos in the system lies in the cross-sectional shape of wires, which can be assumed to be a two-dimensional system. Two-dimensional chaotic systems deformed from a circle have been extensively studied in the quantum chaos community for many years [65]. In a cylindrical NW, the parameters such as height, radius and distance are controlled in order to optimize the transmission, reflection and mode excitation [66, 67].

In a silicon NW array with a stadium-shaped cross-section, broadband absorption is higher by 16.83% compared to that in a cylindrical NW. The finite-difference time-domain (FDTD) method is employed for implementing optical three-dimensional simulations for a stadium-shaped vertical cross-section NW [47].

In the present paper, we discuss the optical absorption of SNW SCs. This is critical since the suggested structure has rudimentary shape compared to alternative NW SCs, while its rate of absorption can be contrasted to that of highly complicated structures.

As to the hurdles of ultra-thin SCs, reduction of the adsorbent constrains their efficiency. For dealing with this problem, diverse methods have been suggested for handling and capturing light within SCs. Such methods usually utilize regular and alternating structures which result in resonance generation at distinct frequencies and this contributes to absorption rate at such frequencies. However, the main drawback of these structures is their low bandwidth, which constrains the level of rise in absorption. In the present article, two methods of enhancing the bandwidth are detailed.

Initially, the impact of irregular structures on NWs is addressed. Observably, the key to the broadband absorption is disorder and irregularity. The approach to reaching top broadband absorption is utilizing more than one NW, with diverse shapes. They can handle numerous cavity modes, while scattering by NWs results in wider absorption spectra. In the case of irregular NW arrays, it is plausible to add to absorption enhancement of SCs without using further absorbing material.

For the next step, few semi-periodic methods are adopted for NW structures and a semi-periodic array is chosen so as to achieve better and more optical antenna effect. The current density can be increased due to compromise between the diffraction and optical antenna effects.

The simulations suggest that cutting the order of NWs results in displaced absorption resonances. As to the optimal design of irregular NWs, the absorption of these structures might be higher than that of the typical structures. The observations suggest that optimized irregular NW arrays have better specifications versus other structures.

In the present study, the impact of horizontally SNW structures on ultra-thin SCs is examined using finite element method (FEM). The cost-effectiveness and easy simulation of the required geometry are the advantages of this method. The specific geometric design approaches demonstrated here lead to achieving broadband absorption in lateral NWs. Finally, the optimization process is carried out to identify the best dimensions of NWs and to maximize the current density of the SCs. Optimization processes can be based on the particle swarm optimization (PSO) algorithm.

## Structure design and simulations

Semiconductor NWs refer to subwavelength optical cavities, a prominent feature of which is supporting resonant modes. To utilize the optical characteristics of NWs it is critical to determine their optical resonances quantitatively via measurements [16, 37, 68–71]. Simulations have manifested how a NW deals with coming light and they can describe thoroughly the characteristics of resonant modes such as profile and amplitude. Measurements, usually photocurrent spectra of individual NW devices, provide support for the simulation and along with calculations they contribute to designing novel NW-based optical cavities.

Nanowire PV devices might include p-type/intrinsic/n-type (p-i-n) dopant modulation for carrier partition in two separate motifs: (1) axial shape with junction along the growth orientation of the NW, and (2) radial shape with a junction between central and coaxial shells. In such structures, the pairs of electrons and holes are made at the moment absorption of photons with energies that are equal to or higher than the bandgap of silicon (*E*_g_ = 1.12 eV for single-crystal silicon). Generation and partition of carriers are highly efficient in the depletion zone because of the built-in field across the p-i-n junction [68, 72, 73]. The electrons (or photo-generated holes) drift if electric field exists and this is done via p-type (or n-type) regions. In the end, they are captured as photocurrent by ohmic contacts made of metal.

The research on laterally assembled silicon NWs is largely based on various regular morphologies that are possible through customized NW synthesis. A variety of silicon NWs can be designed with different morphologies by adjusting the synthetic conditions of NW that allows preferential growth on selected facets [37].

The future nano-electronic devices can be built on silicon NWs. Various techniques have been reported for fabricating silicon NWs. However, the majority of these techniques grow silicon NWs vertically. In the semiconductor industry, integrated circuits are designed and manufactured in a horizontal architecture i.e., the device design is flat compared to the substrate. Once the vertical silicon NWs are introduced in the semiconductor industry, a whole new architecture will be required to fabricate an electronic device. Incorporating these NWs in the current architecture will be more convenient and feasible if the silicon NWs are grown horizontally [35, 74–77].

A proper design of irregular NWs enables the structure to perform better in absorption than regular ones. In addition, previous studies have shown that when multiple NWs with different cross-sectional geometries are used the properties are superior compared to uniform NWs.

**Fig. 1:**
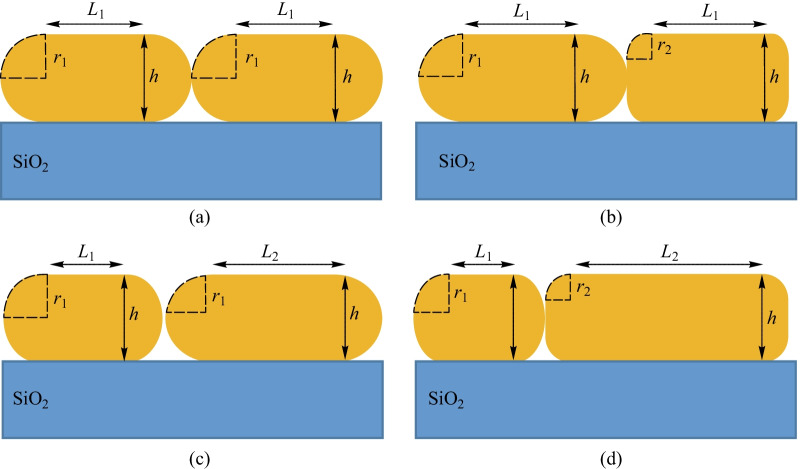
Vertical cross-sectional view of **a** periodic, **b** non-equi radii, **c** nonequilateral, and **d** multiple NW array solar cells

NWs have largely benefitted from different cross-sectional geometries to be fabricated in a variety of forms [42]. However, it is a challenging task to critically control the NW geometry, and the impacts of imprecision of the cross-sectional geometry on the performance of SCs must be taken into account. A two-dimensional system is represented by a stadium shape, where two half-circles of radius *r* and two linear sections of length *L* exist, as shown in Fig. 1.

In the proposed model, the height of all stadium nanowires is set to 200 nm, which indicated the maximum effect of the optical antenna at this thickness. The shape of the NWs had been changed from a hexagon to a circle [78–82] and then to a square [37], while keeping their height fixed at 200 nm.

That SNWs are composed of crystalline silicon is a popular assumption. To optimize simulation, the proposed periodic stadium arrays are placed on a ${\mathrm{SiO}}_{2}$ layer with appropriate thickness. AM1.5 solar spectra are utilized as the radiation spectra. Silicon refractive index at each wavelength and the complex data of the ${\mathrm{SiO}}_{2}$ refractive index are borrowed from Palik’s research [83].

Performance evaluation in different structures needs the absorption spectra and the current density of SCs to be determined. The absorption spectrum per unit volume can be calculated using the intensity of the electric field according to Eq. (1) [84]:

1 $$A = \frac{{\smallint \omega {\text{ Im}}\left( {\varepsilon_{{{\text{Si}}}} } \right)\left| E \right|^{2} {\text{d}}x{\text{d}}y{\text{d}}z}}{{\smallint {\text{Re}}\left[ {E_{0} \times H_{0}^{*} } \right] \cdot n{\text{d}}x{\text{d}}y}}{ },{ }$$

where *ω* is angular frequency, ${E}_{0}$ and ${H}_{0}$ show the electrical and magnetic vectors of the incident wave. *E* is the electrical area within the NWs, *n* is the unit normal vector, and * is the conjugate indicator; *ε*_Si_ is the dielectric power of silicon. Equation (2) models how the *J*_SC_ of the SC can be achieved using the absorption spectrum, under the assumption of inner quantum efficiency [85]:

2 $$J_{{{\text{SC}}}} = \frac{q}{hc}\mathop \smallint \limits_{{\lambda_{{{\text{min}}}} }}^{{\lambda_{{{\text{max}}}} }} \lambda A\left( \lambda \right)I\left( \lambda \right){\text{d}}\lambda .$$

Here, *q* is the charge of the electrons, *λ* is the wavelength of sunlight, *h* is Planck’s constant, *c* is the speed of light, *λ*_min_ and *λ*_max_ are the minimum and maximum wavelengths. $A\left(\lambda \right)$ is the light absorption of the SC and $I\left(\lambda \right)$ refers to the intensity of global solar radiation at AM1.5. These structures are simulated with the FEM [86].

**Fig. 2:**
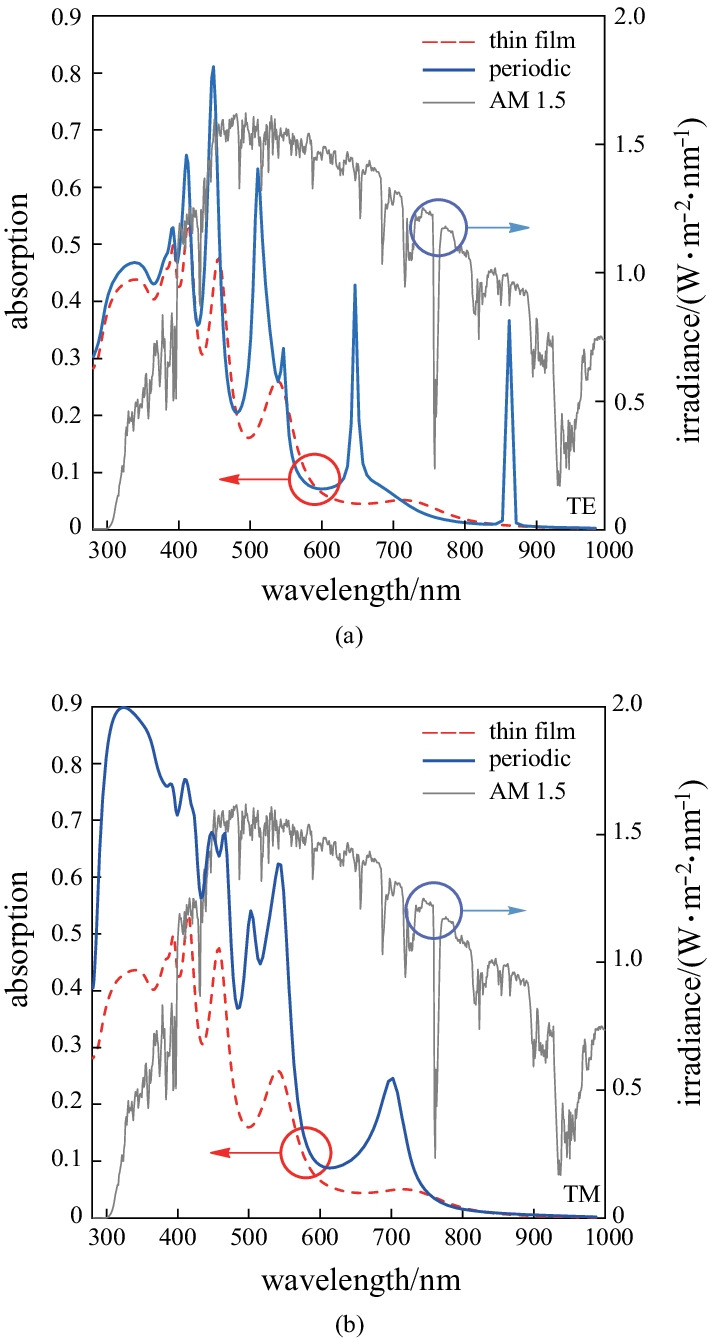
Overlap figure of the absorption intensity of the proposed periodic SNW, thin film SC and the radiation intensity of the sun spectrum (AM1.5) for **a** TE and **b** TM polarization for *h* = 200 nm

In Fig. 1a, the periodic SNW with a height of 200 nm is shown. The absorption spectrum was calculated while the wavelength of a normal plane wave was scanned from 280 to 1000 nm. Transverse-electric (TE) and transverse-magnetic (TM) polarized absorption spectra were calculated for periodic SNW and compared to the thin film NW (Fig. 2). Periodic SNW arrays had the highest absorption for most wavelengths. As shown in Fig. 2a, the broadband absorption created a new resonance at the wavelength of 866.33 nm in the TE polarization, as a result of an optimized design. According to Fig. 2b, at wavelengths of 280–540 nm, absorption peaks were observed at TM polarization.

For a more detailed discussion of this function, the sunlight intensity (AM1.5) is illustrated. There is a sudden decrease in the radiation intensity of the sun for lower wavelengths. Therefore, the absorption intensity for these wavelengths does not have a significant influence on the *J*_SC_ of the SC. In the lateral NW array, the same set of cavity modes shows a single isolated NW. Due to the low-quality of a single NW cavity, there is no additional coupled mode supported by NW array. Thus, if the absorption of light in a single NW is properly tuned by adjusting the morphology or composition of the NW, the array made of these single NWs has the same absorption properties of a single NW. This shows the importance of optical design at the level of individual NWs [82].

In the present paper, the absorption spectra of a periodic SNW array are examined with regard to their cross-sectional morphology. A minor change of morphology of a NW cavity contributes to light absorption adjustment at certain wavelengths, which motivates further development of NW-PVs [37].

One way to reduce the material cost is to use a less-thick SC, which in turn reduces absorption in the thin film. The near band gap absorption, especially for materials of an indirect band gap is feeble for all thin-film SCs. As a result, efforts to improve absorption in thin-film SCs are of particular concern [87].

In this paper, the PSO algorithm is used to get the best parameters. The optimal parameters for the proposed periodic stadium are as follows: ${L}_{1}$ = 83.347 nm and ${r}_{1}$ = 100 nm, which would result in a current density of 6.916 mA/cm^2^ compared to the optimized thin film structure, there is an increase in current density of 66. 89%.

### Effect of changing *r* on current density

**Fig. 3:**
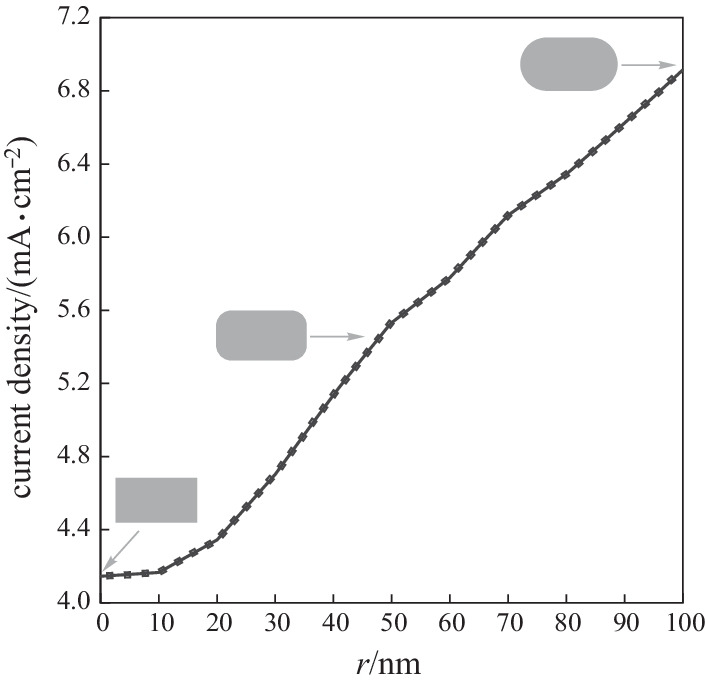
Current density of periodic SNW solar cell as a function of *r*

Parameter *r* is used as a handle to deform a certain geometry, where *r* = 0 indicates the original, undeformed structure and *a* > 0 refers to a proportionally deformed geometry. Given that the NW thickness is 200 nm, the maximum change *r* is 100 nm and no more than this value can be made in the structure of *r*. Figure 3 shows that the increase in the current density of a NW can be explained inherently through considering that a properly shaped deformation is accompanied by the breaking of the symmetry in the structure [88, 89].

### Current density as a function of lateral length

There are two different contributions to the absorption resonances of laterally assembled NWSCs, including localized Fabry–Perot and Whisper Gallery modes. The location and strength of these modes depend heavily on the NW cross-sectional geometry and the lattice constant of the array. Therefore, the change in the NW cross-sectional geometry affects all resonances of the SC.

The silicon NWs’ length is critical to improve efficiency of the SC as larger length of silicon NWs contributes to anti-reflection property. But, at the same time it reduces the SC efficiency. Further studies on surface passivation, device fabrication, and quality enhancement in SiNWs based solar could contribute to a stable and efficient SC device [45].

**Fig. 4:**
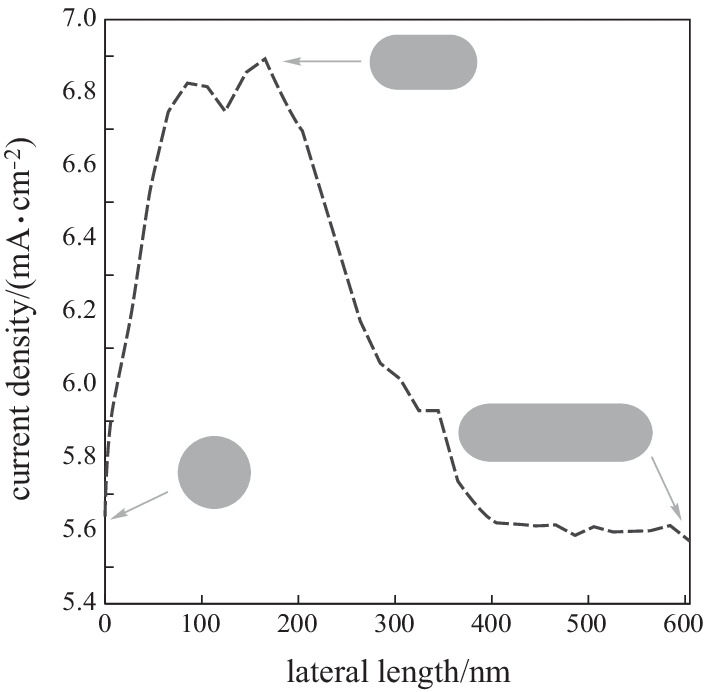
Current density of SNW solar cells as a function of lateral length

For the periodic NW array of the stadium NWs, the current density is shown as a function of the lateral length in Fig. 4. Here the current density is calculated for *r* = 100 nm, *h* = 200 nm as a function of the lateral length from 0 to 600 nm. Since the stadium shape has mirror reflection symmetries with respect to *L* (major axis) and *h* (minor axis), two lights are considered to be linearly polarized along these axes. Since a circle is isotropic, the two spectra of different polarization with a lateral length = 0 nm are the same, i.e., the current density of a stadium cross-section is greater than that of a circular one in NW arrays. The current density with lateral length = 165 nm is 23.21% greater than that at a lateral length of 0 nm.

This significant improvement can only be brought about by the deformation of the wire cross-section since, clearly, deformation is the only parameter that is controlled in computations. It is evident that when NW regularity is broken, the overall current density of the SCs will change. The best current density of the periodic SCs of the SNW array is 6.9 mA/cm^2^.

### Current density of SNW arrays with various cross-sectional morphologies

To study the effects of irregularities on SC performance, it is desirable to start with two rudimentary structures. As seen in Fig. 1b, the cross-sectional view of a SC is made up of non-equi radii SNWs and Fig. 1c the SNWs are non-equilateral. Here *h* refers to the height of the NW, while *r* and *L* are defined as the radius and lateral length of the NW, respectively.

In a non-equi radii SNW, the radius can change from 0 to 100 nm and the lateral length of a non-equilateral is variable. Since the simulations of these structures take a lot of time, obtaining the current density of all possible structures is rather difficult. Therefore, adopting a PSO algorithm can develop the best possible parameters for maximum absorption in these structures.

It is found that the best current density is 7.634 and 7.172 mA/cm^2^ in the non-equilateral structures and non-equi radii SNW SCs, respectively. In each, 10.39% and 3.70% enhancement are secured compared to periodic SNW arrays, respectively.

To increase the absorption enhancement of NWSCs, we can also use multiple NWs with different geometries (Fig. 1d).

In this structure, non-equilateral NWs and non-equi radii have been installed side by side instead of using periodic particle. With PSO algorithm, the best current density obtained for this array is 7.38 mA/cm^2^. This structural change brought about an increase of 2.93% in short circuit current compared to non-equi radii SNWs. Multiple structures can also contribute to elevating SC properties. Cavity modes for TM polarization and whispering-gallery modes for TE polarization lead to an increase in the absorption of the proposed arrays. The sectional geometry of the SNWs determines the strength and position of these modes. Accordingly, modifications in the sectional geometry of the NWs affect all intensities in the SCs.

**Fig. 5:**
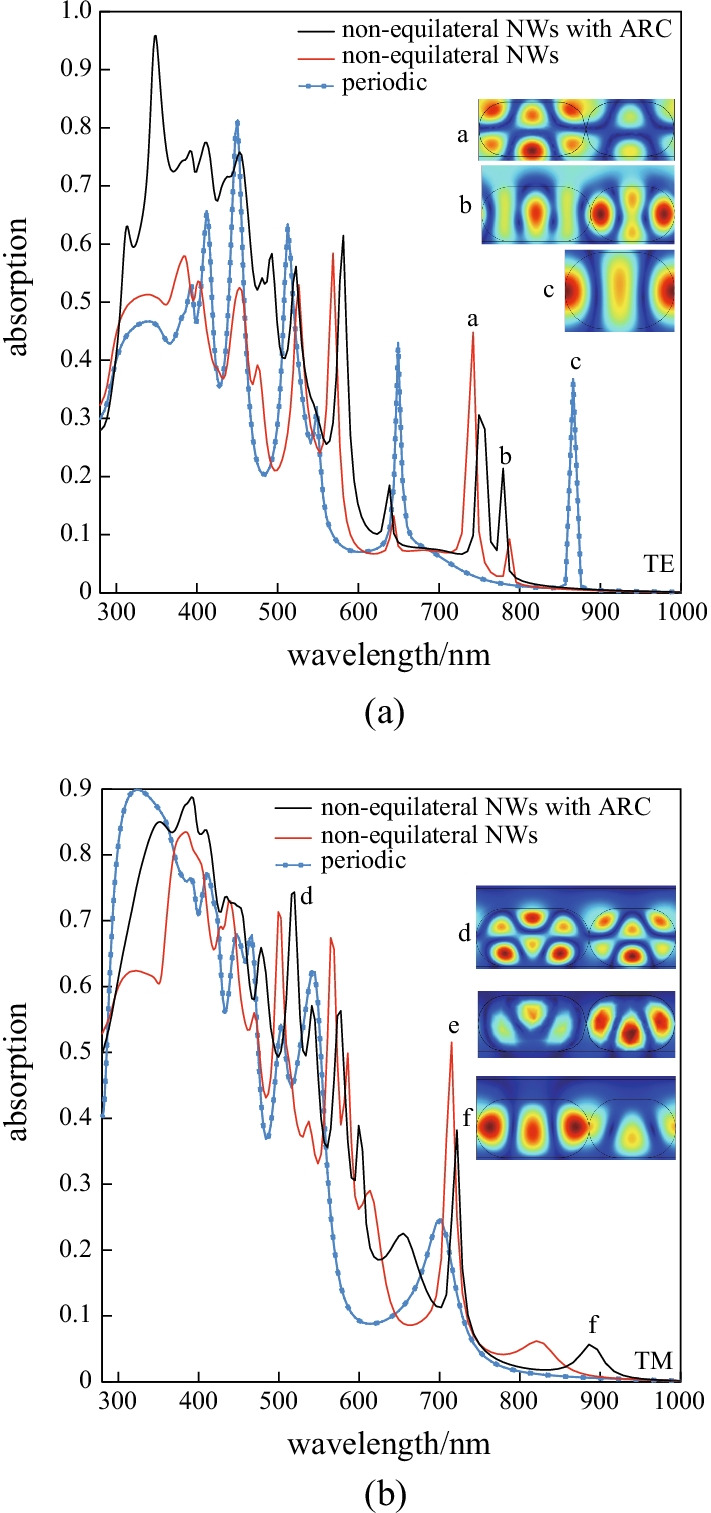
Absorption spectrum of the proposed non-equilateral SNW with/without ARC for **a** TE and **b** TM polarization for *h* = 200 nm

Use of ARC is a promising way to further improve the current density of SCs. Besides, a ${\mathrm{SiO}}_{2}$ coat has covered the upper level of the proposed SNW as an ARC. To evaluate the proposed model, the absorption spectra of the periodic and non-equilateral SNWs array with/without ARC are depicted in Fig. 5.

TE and TM polarizations benefit from a greater absorption in the proposed non-equilateral SNW arrays with ARC, as compared with the non-equilateral SNW arrays without any ARC and with the periodic SNW array. In Fig. 5, the normalized field intensity distributions are calculated with their respective wavelengths to recognize the origin of new resonances. In the case of TE polarization, the normalized field intensity distribution at 742.31, 779.51, and 866.33 nm is depicted in the inset figure a, b, and c of Fig. 5a, respectively. In the case of TM polarization, the normalized field intensity distribution at 515.84, 715.01, and 886.07 nm is depicted in the inset figure d, e, and f of Fig. 5b, respectively.

As shown in Fig. 5, the absorption of non-equilateral SNW arrays with ARC in different structures demonstrates a change in the position and intensity of resonances. Broadening of the spectra is associated with new resonances appearing for non-equilateral SNWs with ARC, caused by the specially designed SNWs. It is also remarkable that absorption enhancement occurs in both polarizations leading to a notable enhancement of current density. Resonant intensities in TE polarization can be even found in the wavelength range of 700–800 nm in Fig. 5a. In addition, a strong resonance in TM polarization at a wavelength of about 700 nm is recorded in Fig. 5b. For TE polarization, however, there is a wavelength range below 400 nm where absorption is stronger than for the other arrays.

The statistics of optimized parameters for the proposed non-equilateral SNW arrays with ARC are as follows: ${L}_{1}$ = 195.7 nm, ${L}_{2}$ = 105.3 nm, ${t}_{\mathrm{arc}}$ = 68.7 nm, and *r* = 100 nm, which results to a current density of 9.301 mA/cm^2^. This structure shows a 21.83% increase in current density versus the optimized non-equilateral SNW arrays without any ARC.

The ARC can be similarly adopted in non-equilateral radii structures and can improve silicon structures and the characteristics of the SC. This work is originally intended for an ideal SC and innovative algorithms are used to optimize the intended structure. It turns out that the multiple SNW array with the ARC structure has the highest current in comparison with the ordinary SNWs structures.

Optimized multiple SNW arrays with the ARC demonstrate a 23.68% increase in short circuit current comparison to those without any ARC.

### Non-close packed SNW array

The maximum diffraction effect and the best absorption efficiency are achieved when NWs are located at a certain distance from each other.

**Fig. 6:**
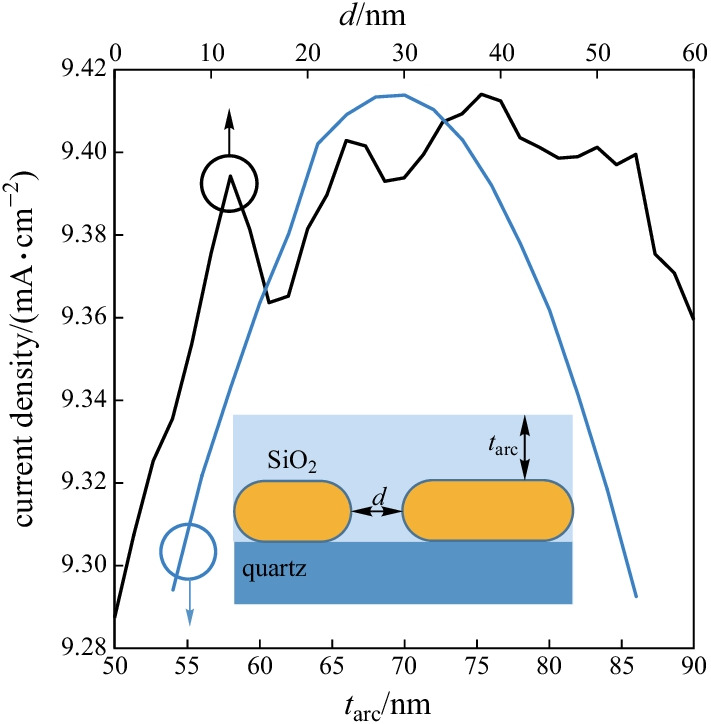
Current density of non-close packed SNWs as a function of *d* and ${t}_{\mathrm{arc}}$

Figure 6 shows the non-close packed SNW arrays while *d* and ${t}_{\mathrm{arc}}$ vary around the optimized values. The non-close packed SNW structure enhances NW absorption in the stadium SCs. Changing the distance between each NW in a non-close packed structure can control the optical antenna effect and diffraction effect. Arrays with non-close packed structure has the highest current density in comparison others.

Since the fluctuations in the current density are quite low, the design is considered robust and there is no need for strict control of NWs’ installation since the proposed structure is not largely dependent on the optimized parameters. SNWs array with *d* = 39.8 nm obtains the maximum *J*_SC_ (9.415 mA/cm^2^).

The enhancement factor in *J*_SC_ at the optimal *d* is approximately 16.11%, compared with that for a close-packed array (periodic with ARC). The *J*_SC_ of a NW array is determined by two sensitive functions of *d*: the interaction of the diffraction effect and the optical antenna effect.

In fact, the optimization process is based on the PSO algorithm so as to investigate the maximum total current density for the non-close packed SNW structures. The obtained optimized parameters consist of the height of ${t}_{\mathrm{arc}}$ set to 67.7 nm. The absorption spectra further improves and the achieved spectrum is almost broadband.

Another finding is the reduction of reflection losses and enhanced absorption efficiency, pointing to the fact that Si${\mathrm{O}}_{2}$ material is a factor that can improve the absorption efficiency in the visible area.

Figure 6 shows that the calculated ${J}_{\mathrm{SC}}$ for ${t}_{\mathrm{arc}}$ at this height is further improved compared with those at other heights (9.415 mA/c${\mathrm{m}}^{2}$). Hence, broadband absorption and *J*_SC_ high values can be achieved by using an optimal ARC in SNW structure.

To determine absorption in these types of SCs, three factors are involved: the optical antenna effect determined by the shape and dimensions of structures, the diffraction effects caused by the periodic nature of the structure, and the extent of NW surface coverage. In this structure, the distance of the NWs is long enough to support an effective interaction with the incident wave irrespective of their geometrical dimensions.

Simulations show that irregularity in NWs leads to the displacement of absorption resonances. If the structure of non-close packed NWs has an optimal design, their absorption is greater than conventional structures. Research suggests that the characteristics of optimized non-close packed NW arrays are superior to other structures.

**Fig. 7:**
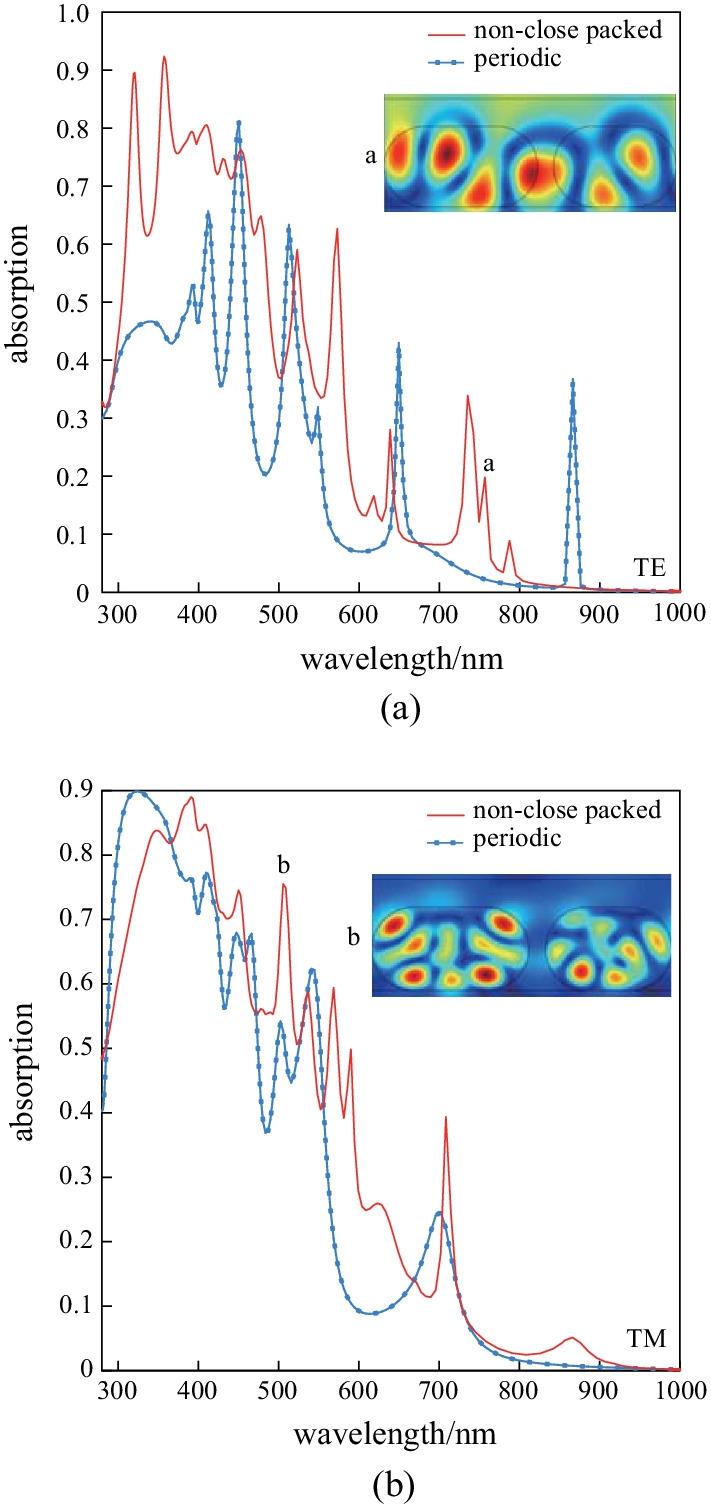
Absorption spectrum of the proposed non-close packed SNW arrays for **a** TE and **b** TM polarization for *h* = 200 nm

As shown in Fig. 7, various resonances are observed at a wavelength of 400–600 nm. This can be the result of the compromise between the optical antenna and the diffraction effects. Resonances at 756.75 nm for TE polarization and 505.78 nm for TM mode are shown in Fig. 7a and 7b respectively.

In this structure, the interaction between NWs leads to a displacement of resonance compared to the periodic structure. However, thinner NWs could benefit more from non-close packed SNW arrays, where the effects of the optical antenna are relatively high.

In contrast, TM polarization enables the periodic SNW to have a better performance than the non-close packed one. This observation is also in line with other findings in Refs. [90, 91]. The absorption of the proposed arrays was enhanced by cavity modes for TM polarization and whispering-gallery modes for TE polarization.

### Effects of incident angle

**Fig. 8:**
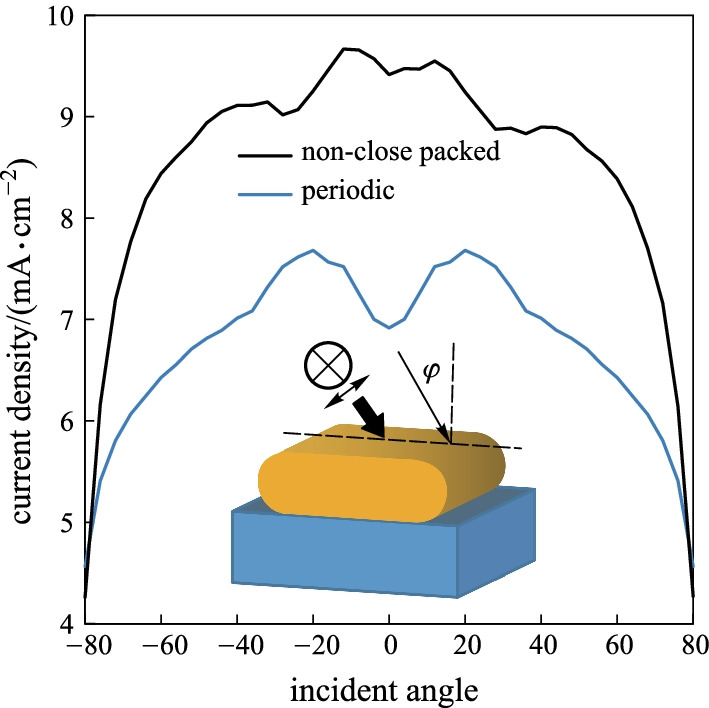
Incident angle for periodic SNW without ARC and non-close packed SNW array

As the last step, an analysis of the incident light angle affect is given in Fig. 8. Here, the angle is swept from − 80° to + 80° for non-close packed and periodic SNW array. The asymmetrical design of the NWs resulted in a nonsymmetric response of non-close packed arrays. However, the current density of non-close packed arrays is more significant for all incident angles than the periodic arrays.

As shown in Fig. 8, when the current density is compared in a certain angle of incidence with that at oblique angles, we see an improvement which can be explained by polarization (Brewster) angle effect. In this angle, the light is perfectly transmitted into the absorbing material without any reflection. On the other hand, the increment obtained in path length of incoming light can also be a second cause. The simulation results maintain that the current density of the non-close packed SNW with ARC is better than the periodic SNW incident cases for incidence angles between − 80° and + 80°. It is noteworthy that against expectation the top current density enhancement is not achieved at normal incidence. This could be explained by optimization of the geometric parameters (due to computational resources).

Table 1 lists the results of *J*_SC_ and the related enhancements of different structures through the PSO algorithm, under the assumption that *h* = 200 nm.

**Table 1:** Optimized parameters and total current densities for different structures

Structure	SNW without ARC				
	Thin film	Periodic	Non-equi radii	Multiple	Non-equilateral
*L*_1_/nm	284.350	83.347	174.0	162.17	188.9
*L*_2_/nm	–	–	–	238.14	121.8
$r$_1_/nm	–	100	99.4	95.5	100
$r$_2_/nm	–	–	76.3	98.6	–
$t$_arc_/nm	–	–	–	–	–
*d*/nm	–	–	–	–	–
*J*_SC_/(mA·cm^−2^)	4.1441	6.916	7.172	7.3820	7.6346
Enhancement /%	–	66.89	73.07	78.13	84.23

Structure	SNW with ARC				
	Periodic	Non-equi radii	Multiple	Non-equilateral	Non-close packed
*L*_1_/nm	181.9	163.6	184.4	195.7	178
*L*_2_/nm	–	–	102.7	105.3	104.6
$r$_1_/nm	100	98.8	94.1	100	100
$r$_2_/nm	–	91.6	98.3	–	100
$t$_arc_/nm	67.9	70.0	66.5	68.7	67.7
*d*/nm	–	–	–	–	39.8
*J*_SC_/(mA·cm^−2^)	8.109	9.0380	9.130	9.301	9.415
Enhancement /%	95.68	118.09	120.31	124.44	127.19

## Conclusions

In the present paper, the effects of geometrical shape and structural order of the laterally assembled stadium silicon NWs on the performance of ultra-thin SCs are studied.

This study shows that modifying geometry can enhance the functionality of geometry, that is, without needing more materials or incurring a higher cost. Moreover, it is concluded that the relaxed tolerances of a chaotic system make fabrication easier. The proposed different methods to design a broadband NWSC are: first, using the arrangement of NWs without any ARC, and second, employing multiple NWs in different shapes and geometries with ARC. NWs can be used both horizontally and vertically. With the vertical method, no precise control is exerted on the dimensions and arrangement of the NWs. In the horizontal method, however, NWs can be manufactured with high precision on a suitable substrate. In addition, the horizontal method makes it possible to collect charge carriers radially, leading to higher SC efficiency.

The next point addressed here is the effect of irregularity on the performance of assembled stadium silicon NWSCs. The interaction between the optical antenna and diffraction effects can also lead to a better and greater absorption of non-close packed arrays compared to the periodic arrangement.

In these proposed structures, an optimization process is conducted to obtain the maximum current density. Observations shows that the optimized non-close packed NW arrays characteristics are superior to other structures. Overall, 127.19% enhancement in current density is obtained by optimizing the non-close packed NW arrays.
